# Detection of SARS-CoV-2 Variants Mu, Beta, Gamma, Lambda, Delta, Alpha, and Omicron in Wastewater Settled Solids Using Mutation-Specific Assays Is Associated with Regional Detection of Variants in Clinical Samples

**DOI:** 10.1128/aem.00045-22

**Published:** 2022-04-05

**Authors:** Marlene Wolfe, Bridgette Hughes, Dorothea Duong, Vikram Chan-Herur, Krista R. Wigginton, Bradley J. White, Alexandria B. Boehm

**Affiliations:** a Gangarosa Department of Environmental Health, Rollins School of Public Health, Emory University, Atlanta, Georgia, USA; b Verily Life Sciences, South San Francisco, California, USA; c Civil and Environmental Engineering, University of Michigan, Ann Arbor, Michigan, USA; d Civil and Environmental Engineering, Stanford University, Stanford, California, USA; University of Nebraska-Lincoln

**Keywords:** SARS-CoV-2, Omicron, wastewater, COVID-19, Delta, epidemiology

## Abstract

Changes in the circulation of SARS-CoV-2 variants of concern (VOCs) may require changes in the public health response to the COVID-19 pandemic, as they have the potential to evade vaccines and pharmaceutical interventions and may be more transmissive than other SARS-CoV-2 variants. As such, it is essential to track and prevent their spread in susceptible communities. We developed digital reverse transcription (RT)-PCR assays for mutations characteristic of VOCs and used them to quantify those mutations in samples of wastewater settled solids collected from a publicly owned treatment works (POTW) during different phases of the COVID-19 pandemic. Wastewater concentrations of single mutations characteristic of each VOC, normalized by the concentration of a conserved SARS-CoV-2 N gene, correlate with regional estimates of the proportion of clinical infections caused by each VOC. These results suggest that targeted RT-PCR assays can be used to detect variants circulating in communities and inform the public health response to the pandemic.

**IMPORTANCE** Wastewater represents a pooled biological sample of the contributing community and thus a resource for assessing community health. Here, we show that emergence, spread, and disappearance of SARS-CoV-2 infections caused by variants of concern are reflected in the presence of variant genomic RNA in wastewater settled solids. This work highlights an important public health use case for wastewater.

## INTRODUCTION

During an infectious disease outbreak, it is critical to detect cases quickly and to estimate the extent and timing of the outbreak to target interventions to mitigate spread. The detection of targets associated with infectious agents in wastewater can be used to infer information on the health of an entire population and provide critical outbreak monitoring services. This technique has been used widely during the COVID-19 pandemic, as SARS-CoV-2 RNA is readily detectable in wastewater and concentrations of RNA correlate with laboratory-confirmed COVID-19 infections in the contributing communities (1–4). Wastewater has previously been used to track gastrointestinal infections, including poliovirus (5), and this work has been extended to track not only COVID-19 (6) but also other respiratory viruses such as respiratory syncytial virus (RSV) (7). Using wastewater to track community health has the advantage of providing information on an entire community without relying on individual clinical testing, which may be expensive or unavailable and requires individuals to alter their behavior to seek testing. Wastewater may be a leading indicator of community health when shedding by infectious individuals precedes symptom onset.

The COVID-19 pandemic has seen SARS-CoV-2 acquire mutations that have given rise to variants with distinguishing characteristics. Variants of concern (VOCs) or interest (VOIs) are variants that may evade vaccines or other pharmaceutical interventions, be more transmissible, or cause more severe illness. Variant classifications by the World Health Organization (WHO) and the U.S. Centers for Disease Control and Prevention (CDC) have changed over the course of the pandemic, but VOCs are named according to the Greek alphabet and have included Alpha, Beta, Gamma, Delta, Lambda, Mu, and Omicron (8, 9). The emergence of variants is primarily identified by sequencing of clinical specimens; this same approach is then typically used to track the spread of VOCs into and throughout communities. A health department or clinical laboratory will choose a subset of all specimens to sequence, and results are usually available within 2 weeks. These data could lack community representation if samples from some clinics are more likely to be sequenced than others or may be biased when specific samples are chosen for sequencing because of patient characteristics. A 2-week processing time may prevent a fast public health response to a spreading variant of concern.

Monitoring variants in wastewater may overcome some of the problems with relying on sequencing clinical specimens to track variant emergence and spread. A wastewater sample is representative of the entire contributing community and therefore lacks bias that is common for sequencing of clinical specimens. However, a wastewater sample is more complex than a clinical specimen: it contains many different types of viruses (10) that have undergone different degrees of degradation (11). Sequencing SARS-CoV-2 RNA from wastewater likely requires enrichment or amplification of the SARS-CoV-2 genome (12). An alternative approach for variant tracking in wastewater is application of targeted reverse transcription (RT)-PCR assays that amplify and allow detection of short genomic sequences characteristic of the variant.

Several publications to date have explored the use of targeted assays to detect SARS-CoV-2 variants in wastewater. Heijnen et al. (13) applied a commercial digital RT-PCR assay to wastewater influent samples to detect a single nucleotide polymorphism (SNP) (mutation N501Y) present in Beta and Alpha. Lee et al. (14) and Graber et al. (15) applied RT-qPCR assays that detect mutations present in Alpha to wastewater samples. Yaniv et al. developed RT-qPCR assays for Gamma and Delta (16) and for Alpha and Beta (17) and applied them to 4 and 10 wastewater samples, respectively, as proof of concept. To date, there is limited research (18, 19) to apply targeted assays for characteristic mutations of diverse variants to wastewater samples across different phases of the pandemic to identify emergence patterns and compare those to data from variants in clinical specimens.

The present study developed novel targeted digital droplet RT-PCR (ddRT-PCR) assays for the detection of six characteristic mutations from distinct variants in wastewater. In particular, we developed and utilized assays for mutations characteristic of Alpha, Beta and Gamma (Beta/Gamma), Delta, Mu, Lambda, and Omicron and then measured these in wastewater solids from a publicly owned treatment work (POTW) located in the Bay Area of California, USA. We measured concentrations in wastewater settled solids, as concentrations of SARS-CoV-2 RNA are enriched several orders of magnitude in solids relative to those in liquid wastewater (20, 21). We subsequently compared the measurements to data on occurrence of those variants in clinical specimens, aggregated at the state level.

## RESULTS

### Lambda, Mu, and Beta/Gamma variant mutation assay specificity.

**FIG 1:**
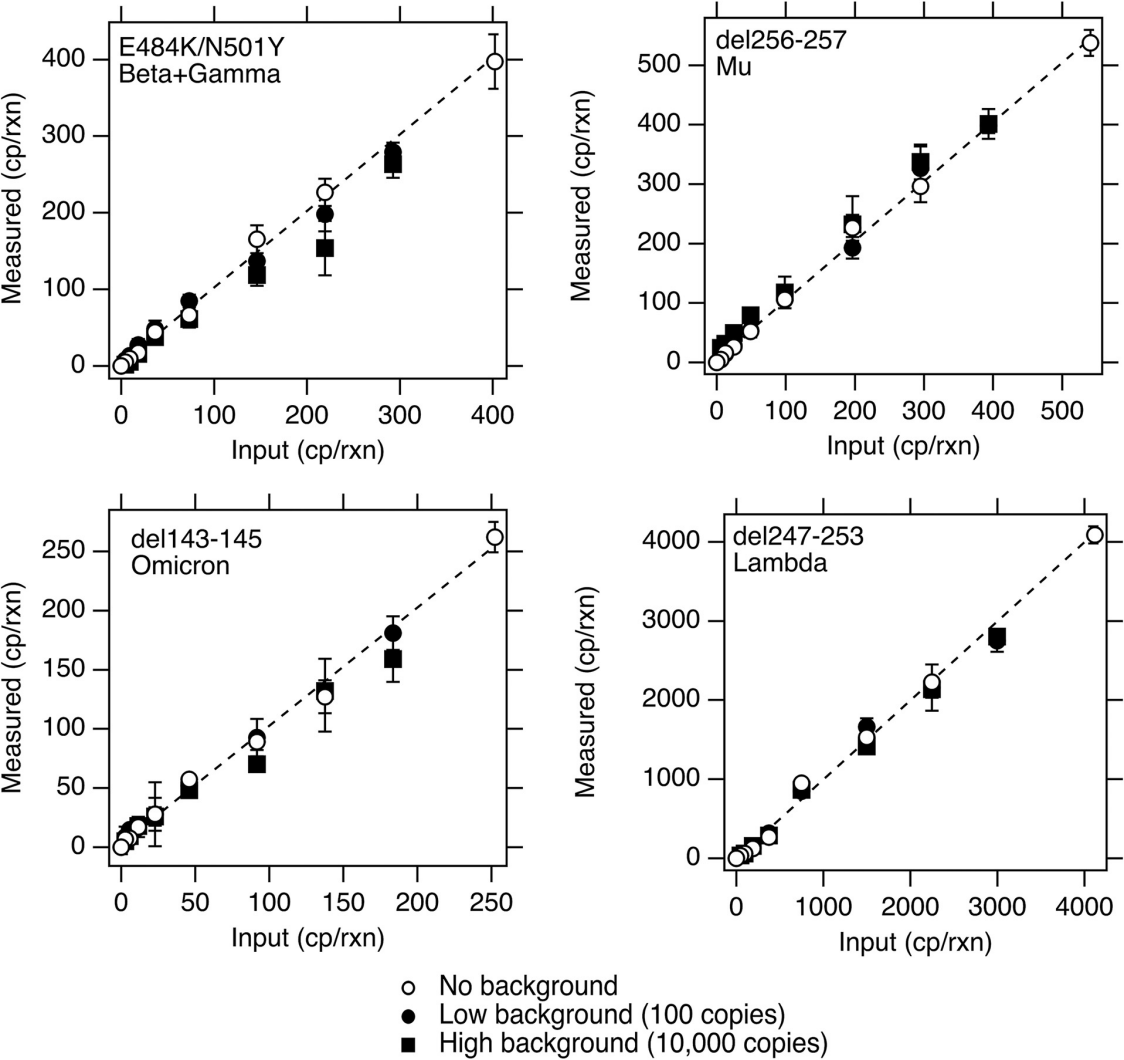
Copies (cp) of mutations measured when RNA containing the mutation was diluted into no, low, or high background of WT gRNA. Low background is 100 copies/well, and high background is 10,000 copies/well, where “copies” refers to copies of genomes of WT gRNA. Markers show the average across three replicate wells, and error bars represent standard deviations. In some cases, the error bar is not visible because it is smaller than the marker. rxn, reactions equivalent to one well.

*In silico* analysis of the Lambda, Mu, Beta/Gamma, and Omicron variant mutation assays indicated no cross-reactivity between the variant mutation assays and deposited sequences in NCBI. When challenged against the respiratory virus panel and genomic RNA (gRNA) from wild-type (WT) SARS-CoV-2 and other variants (Table 1), no cross-reactivity was observed. When mutation assays were tested using their target variant gRNA diluted in a background of high and low WT SARS-CoV-2 RNA, there was no evidence of cross-reactivity (Fig. 1). Positive and negative controls run on all the ddPCR plates were positive and negative, respectively. These results suggest that the variant mutation ddRT-PCR assays are specific. Yu et al. (19) provide details on the specificity and sensitivity of the Alpha and Delta mutation assays, which are also specific and sensitive.

**TABLE 1:** Details of variants and their characteristic mutations included in this study^*a*^

Variant name(s)	Mutation(s) (gene location)	% of variant genomes, observed globally, with mutation(s) in GISAID as of 10 Jan 2022 (total no. of variant genomes with mutations/total no. of variant genomes)	Positive control used in sensitivity testing	SARS-CoV-2 genomes tested against *in vitro* for specificity
Alpha	HV69-70 (del69-70) (S gene)	97% (1,106,137/1,143,476)	Reported by Yu et al. (19)	Reported by Yu et al. (19)
Delta	del156-157/R158G (S gene)	92% (3,644,016/3,953,372)	Reported by Yu et al. (19)	Reported by Yu et al. (19)
Beta/Gamma	E484K/N501Y (S gene)	Gamma, 94% (112,925) Beta, 85% (34,576/40,553)	Positive clinical swab sequenced as P.1 (Gamma)	WT gRNA and Alpha
Mu	del256-257 (ORF3a)	95% (13,978/14,712)	Positive clinical swab from Stanford sequenced as B.1.621	WT gRNA, Alpha, Beta, Delta, and Gamma
Lambda	del247-253 (S gene)	84% (8,029/9,577)	gRNA from cultivated Lambda variant from Pinsky Lab C.37	WT gRNA, Alpha, Beta, Gamma, Delta, and Mu
Omicron	del143-145 (S gene)	95% (212,997/224,673)	Synthetic Omicron gRNA from Twist control 48	WT gRNA, Alpha, Beta, Gamma, Delta, Mu, and Lambda

a The table includes the variant names, the characteristic mutations for which the ddRT-PCR assays were developed, the percentages of variant genomes with the characteristic mutation(s), the positive controls used in the sensitivity testing experiments, and the SARS-CoV-2 genomes that were used, along with the respiratory panel, in the specificity testing conducted *in vitro*.

### Variant mutation RNA in wastewater.

All positive and negative controls were positive and negative, respectively, indicating that assays performed well and without contamination. Bovine coronavirus (BCoV) recoveries were higher than 10%, and pepper mild mottle virus (PMMoV) concentrations were within the expected range for the POTW, suggesting an efficient and acceptable recovery of RNA during RNA extraction (see Fig. S1 in the supplemental material).

**FIG 2:**
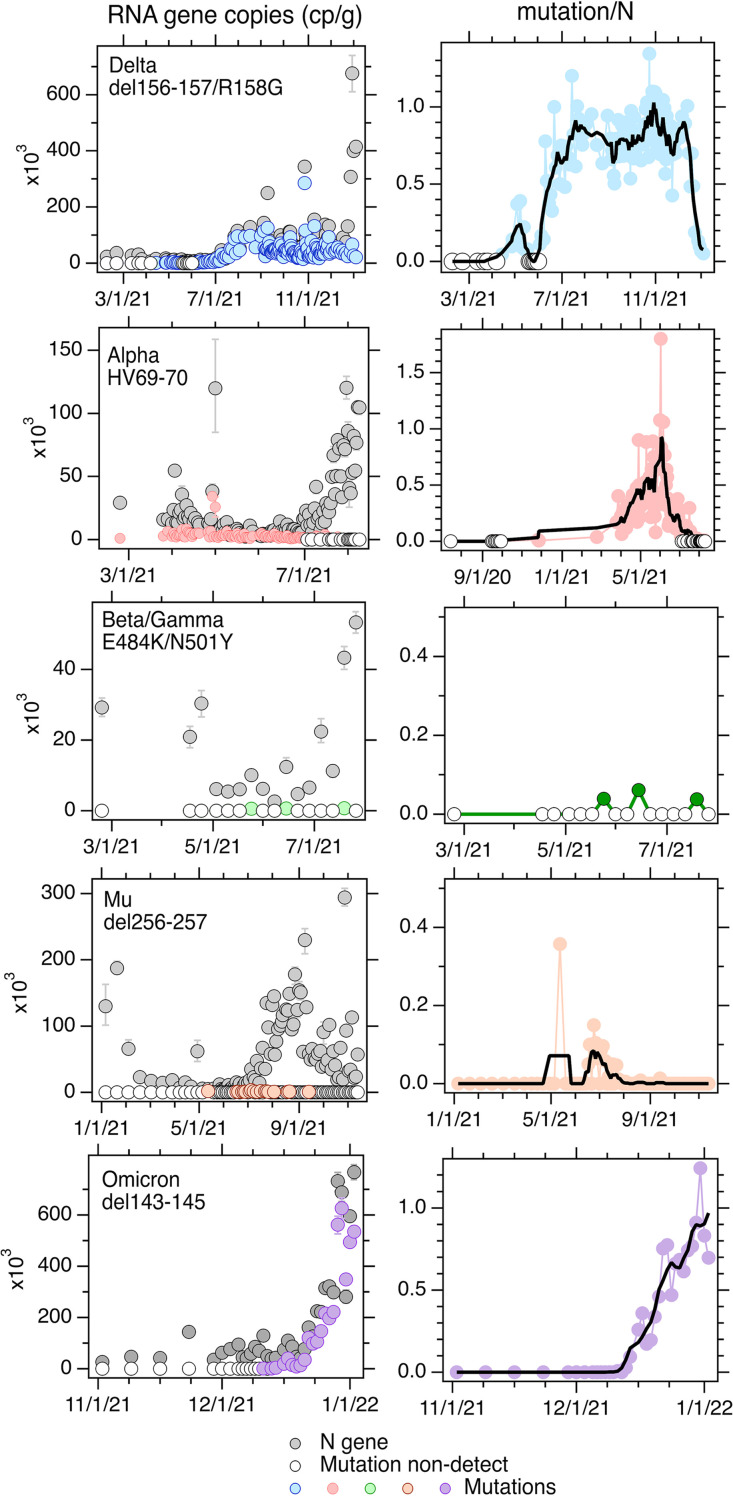
Left column, concentrations (copies [cp] per gram dry weight) of the N gene and the indicated mutation in wastewater solids as a function of time. Open circles indicate nondetections of the mutation gene. Error bars represent standard deviations and include Poisson error and replicate well error; these data were output from the ddPCR machine software as “total error.” Right column, the concentration of the mutation normalized by the concentration of the N gene as a function of time (relative mutation concentration, unitless). The black line represents the 5-point smoothed value for the dates. Open circles are nondetections. Nondetections are shown as 0 on the plots. The Alpha mutation data are from a report by Yu et al. (19); the Delta mutation data through 31 July 21 are from the same report (19). Dates on the figure are given as month/day/year.

As described previously by Yu et al. (19), the Alpha mutation was not detected in wastewater solids prior to January 2021. After that time, it was detected at low relative concentrations until late March 2021, when its relative concentration started to increase until early June 2021, at which time its relative concentration peaked. The concentration began to decrease until the mutation became undetectable in late June 2021 (Fig. 2).

The Delta mutation was not detected in wastewater solids until early April 2021, at which time it increased and was detectable for about a month before it fell to nondetectable levels again for 2 weeks. Thereafter, the concentration of the Delta mutation rose over the month of June until it was present at about the same concentration as the N gene; thereafter, the concentration stayed approximately equivalent to the N gene until the beginning of December 2021 (Fig. 2). The relative concentration subsequently decreased until the end of December 2021.

The Omicron mutation was absent in the samples tested prior to 11 December 2021. After first detection on 11 December, the concentrations rose steadily until the relative concentration was close to 1 at the end of December (Fig. 2).

The mutation present in Beta/Gamma was rarely detected in wastewater solids (Fig. 2). It was not detected in wastewater until late May 2021, when it was detected at a very low relative concentration. It was detected a total of three times between late May 2021 and the end of July 2021, all at low concentrations relative to the N gene.

The mutation present in Mu was not detected until May 2021, when it was detected at a fairly high concentration relative to the N gene in a single sample. Thereafter, it was not detected again until mid-June, after which its relative concentration increased for 1 month until the beginning of July. It then decreased over the following month until the beginning of August, after which the mutation was no longer detected (Fig. 2). The Lambda mutation assay was applied to 2 samples in November 2021 and was not detected.

**FIG 3:**
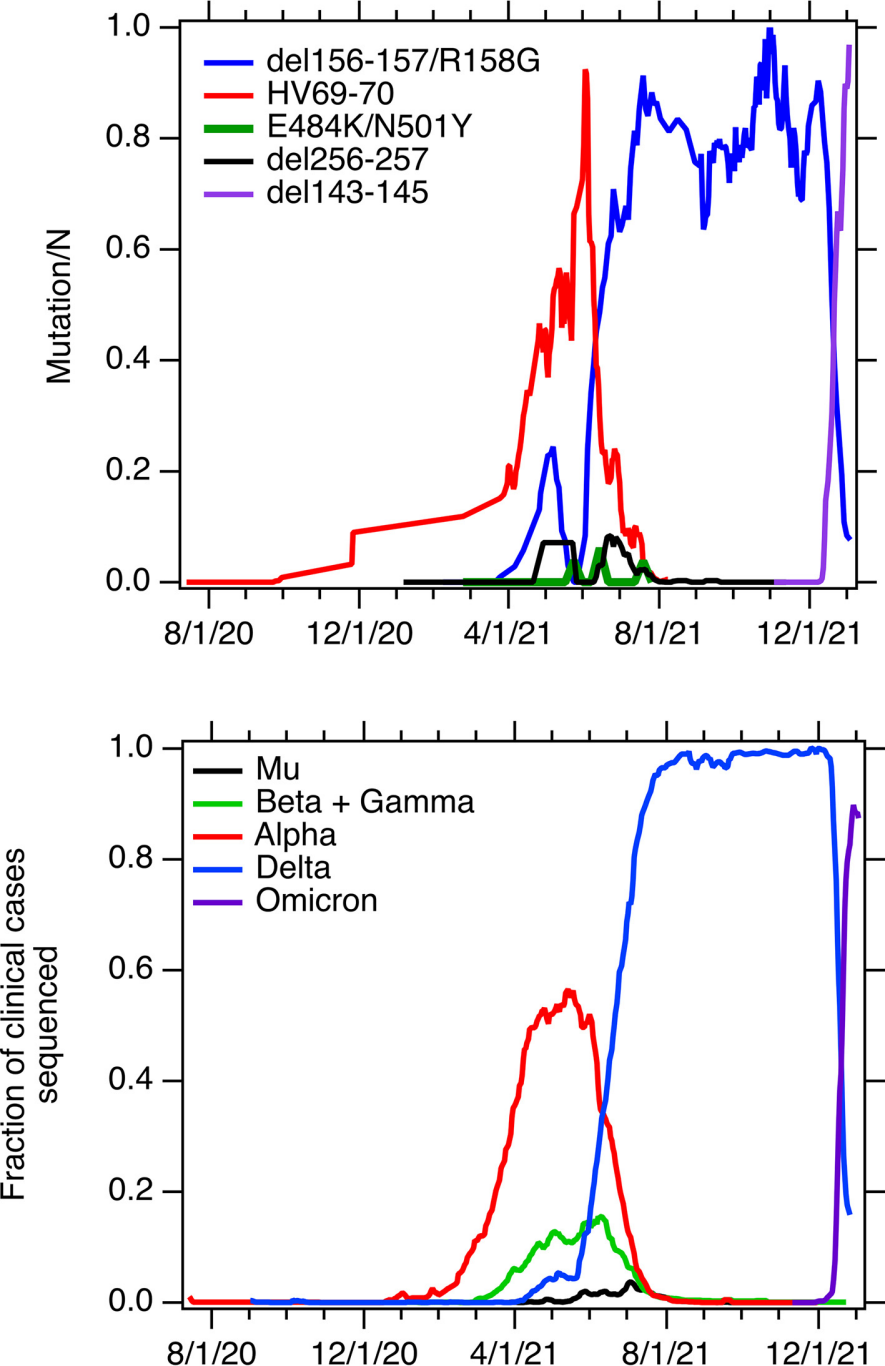
Top graph, five-point smoothed relative concentrations of mutations in wastewater solids (unitless) with the exception of that for E484K/N501Y, which are the raw data; nondetections were taken as 0. Bottom graph, the fraction of all sequenced clinical specimens in California that were classified as the indicated variant (7-day rolling average from https://www.outbreak.info). Dates on the figure are given as month/day/year.

The 5-sample smoothed relative concentrations of the Alpha, Delta, Mu, and Omicron mutations and the raw relative concentrations of the Beta/Gamma mutations are shown in Fig. 3 along with the 7-day rolling average fraction of clinical specimens from California assigned as each variant. The temporal trends in the relative wastewater concentrations and clinical specimen data are qualitatively similar. The wastewater variant mutation data (raw data, Fig. 2) are positively, significantly associated with the clinical variant data (tau = 0.75, *P* < 10^−15^ for Alpha; tau = 0.42, *P* < 10^−13^ for Delta; tau= 0.91, *P* < 10^−13^ for Omicron; tau = 0.36, *P* < 10^−4^ for Mu) with the exception of data for Beta/Gamma. The relative concentration of the Beta/Gamma mutation was positively associated with the fraction of clinical specimens assigned as Beta and Gamma (tau = 0.14, *P* = 0.5), but the association was not statistically significant. This may be due to the relatively low cadence of measurements, as we measured the mutation only once per week; this is low compared to the frequency of variability typically observed in wastewater measurements (1). There was no reported case of Lambda in the state from November 2021, and our lack of detection of the Lambda mutation in that month is consistent with this. The positive associations between relative variant mutation concentrations and the fraction of clinical specimens assigned to Alpha and Delta are consistent with findings described by Yu et al. (19) using sewershed-aggregated clinical data over a different time period.

## DISCUSSION

Wastewater results are indicative of the replacements of consecutive variants in circulation over time. The decline in relative wastewater concentrations of the Alpha mutation is coincident with the rise in relative wastewater concentrations of the Delta mutation, suggestive of Delta outcompeting Alpha in causing infections in susceptible populations. Beta and Gamma mutations began to appear in wastewater along with Mu mutations as the relative concentration of the Delta mutation was rising. It appears that these variants were also present but not able to compete with Delta, as their relative concentrations decreased to nondetection shortly after their appearance in wastewater. The increase in relative wastewater concentrations of the Omicron mutation is coincident with the decline in the relative concentrations of the Delta mutation, suggesting that Omicron potentially outcompeted Delta or that a large increase in Omicron incident cases occurred atop a stable background of Delta incident cases.

Several other studies have reported agreement between detection of characteristic variant mutations in wastewater and the occurrence of variants in clinical specimens. Lee et al. (14) reported a 3-fold-higher increase in a characteristic mutation in Alpha in wastewater from January to March 2021, comparable to an increased fraction in Alpha sequences from clinical samples deposited in GISAID during the same time period. Graber et al. (15) reported agreement between wastewater trends of a characteristic mutation from Alpha and data aggregated at the city level on Alpha circulation based on clinical specimens. Yaniv et al. (18) reported lack of detection of a characteristic mutation in Alpha in wastewater when clinical data suggested that it was not circulating.

The clinical data used in this study are imperfect. The fraction of sequenced clinical specimens assigned to each variant may be biased by the selection of specimens to sequence and the number of specimens sent for sequencing. The data displayed in Fig. 3 are aggregated across the state and may not reflect the occurrence of infections caused by different variants in the population contributing to the sewershed and represented in the wastewater data, particularly for variants with low occurrence rates. Despite these limitations, the wastewater variant mutation measurements correlate well with the variant clinical data.

SARS-CoV-2 RNA in wastewater is a complex mixture of gRNA of all circulating variants in a given community. SARS-CoV-2 gRNA present in wastewater may be present in an intact or damaged viral capsid with or without an envelope (22) and may have undergone damage or fragmentation (11). In contrast, a clinical specimen contains numerous copies of one SARS-CoV-2 variant, with the gRNA likely intact. Given the complexity of wastewater SARS-CoV-2 gRNA, the presence of a single characteristic mutation in wastewater cannot definitively indicate that a variant is present, because a variant is defined by the presence of multiple mutations on a single genome. A single characteristic mutation detected in a wastewater sample could theoretically be from a different variant, known or unknown, containing the same mutation. Even the detection of two mutations characteristic of a specific variant in wastewater does not prove that the variant is present, because those two mutations could have originated from different genomes. Moreover, the characteristic mutations used in this study are not present in 100% of the associated variant genomes. Despite these limitations, our results suggest that the concentration of a single mutation characteristic of a variant of concern relative to the concentration of a conserved SARS-CoV-2 target (the N gene) is associated with the proportion of regional infections caused by the variant.

These findings suggest that for variants of concern, valuable insights are available into the circulation of the variants through the use of wastewater, and these insights are attainable using assays that target a single characteristic variant mutation. Development of assays for SARS-CoV-2 variants requires *in silico* assay design, procurement of primers, probes, and positive-control material, and specificity and sensitivity testing. The rate-limiting step in this process, we have found, is the procurement process. Targeted ddRT-PCR assays can be applied to samples with a turnaround time for results of less than 24 h, and new targeted assays can be quickly developed and applied to wastewater when new variants are identified and expected to spread into communities to gain insight into their local emergence. We were able to implement this process in real time for development and implementation of the Omicron mutation assay, which we were able to apply to daily samples at this POTW starting 6 December 2021 to capture the emergence of the variant at high resolution.

## MATERIALS AND METHODS

### Assay development.

Assays were designed to target mutations characteristic of the following variants: Alpha (HV69-70), Delta (del156-157/R158G), Beta and Gamma (together) (E484K/N501Y), Mu (del256-257), Lambda (del247-253), and Omicron (del143-145) (Table 1). These characteristic mutations were chosen because they are present in high percentages in the associated variant sequences in GISAID (Table 1, information accessed via https://www.outbreak.info), and they represent deletions or multiple single nucleotide polymorphisms (SNPs) in close proximity and thus are likely to be more specific than assays targeting a SNP. Assays were developed *in silico* using Primer3Plus (https://primer3plus.com/). Mutation and adjacent sequences were obtained from genomes downloaded from NCBI. The parameters used in assay development (that controlled sequence length, GC content, and melt temperatures) are provided in Table S1 in the supplemental material. Primers and probe sequences are provided in Table 2. The development and testing of the HV69-70 and del156-157/R158G mutation assays are reported elsewhere (19), so additional details are not provided on these assays herein.

**TABLE 2:** Primer and probe sequences used in this study to target characteristic mutations in variants

Target^*a*^	Primer/probe	Sequence
N gene	Forward	CATTACGTTTGGTGGACCCT
	Reverse	CCTTGCCATGTTGAGTGAGA
	Probe	CGCGATCAAAACAACGTCGG (5′ FAM/ZEN/3′ IBFQ)
BCoV	Forward	CTGGAAGTTGGTGGAGTT
	Reverse	ATTATCGGCCTAACATACATC
	Probe	CCTTCATATCTATACACATCAAGTTGTT (5′ FAM/ZEN/3′ IBFQ)
PMMoV	Forward	GAGTGGTTTGACCTTAACGTTTGA
	Reverse	TTGTCGGTTGCAATGCAAGT
	Probe	CCTACCGAAGCAAATG (5′ HEX/ZEN/3′ IBFQ)
HV69-70 (Alpha)	Forward	ACTCAGGACTTGTTCTTACCT
	Reverse	TGGTAGGACAGGGTTATCAAAC
	Probe	ATGCTATCTCTGGGACCAAT (5′ FAM or HEX/ZEN/3′ IBFQ)
E484K/N501Y (Beta and Gamma)	Forward	CTGAAATCTATCAGGCCGGT
	Reverse	GTTGGTAACCAACACCATAAG
	Probe	CACACCTTGTAATGGTGTTAAAGGTT (5′ FAM or HEX/ZEN/3′ IBFQ)
del156-157/R158G (Delta)	Forward	ATTCGAAGACCCAGTCCCTA
	Reverse	AGGTCCATAAGAAAAGGCTGA
	Probe	TGGATGGAAAGTGGAGTTTATTCTAG (5′ FAM or HEX/ZEN/3′ IBFQ)
del256-257 (Mu)	Forward	CAAATTCACACAATCGACGGT
	Reverse	GTCGTCGTCGGTTCATCATA
	Probe	TCATCCGGAGTTATCCAGTAATGG (5′ FAM or HEX/ZEN/3′ IBFQ)
del247-253 (Lambda)	Forward	TCGGCTTTAGAACCATTGGT
	Reverse	TCAAGTGCACAGTCTACAGC
	Probe	TGCTTTACATAATTCTTCTTCAGGTTGGAC (5′ FAM or HEX/ZEN/3′ IBFQ)
del143-145 (Omicron)	Forward	ATTCGAAGACCCAGTCCCTA
	Reverse	ACTCTGAACTCACTTTCCATCC
	Probe	TTGTAATGATCCATTTTTGGACCACAA (5′ FAM or HEX/ZEN/3′ IBFQ)

a The variant containing the characteristic mutation is shown in parentheses after the name of the targeted mutation. Information on the fluorescent molecule and quenchers used for the probes are provided in parentheses after their sequence. FAM, 6-fluorescein amidite; HEX, hexachloro-fluorescein; ZEN, a proprietary internal quencher from IDT; IBFQ, Iowa Black FQ.

### Specificity screening against other targets.

Primers and probe sequences were screened for specificity *in silico* using NCBI BLAST and then tested *in vitro* against a virus panel (NATtrol respiratory verification panel, NATRVP2-BIO; Zeptomatrix) that includes several influenza and coronavirus viruses, “wild-type” gRNA from SARS-CoV-2 strain 2019-nCoV/USA-WA1/2020 (ATCC VR-1986D), which does not contain the mutations (here referred to as WT gRNA), and a combination of heat-inactivated SARS-CoV-2 strain B.1.1.7 (SARS-CoV-2 variant B.1.1.7, ATCC VR-3326HK), a positive clinical sample confirmed as Mu provided by Ben Pinsky at Stanford Virology Laboratory, and synthetic gRNA from Twist Biosciences (South San Francisco, CA, USA) for Beta (Twist control 16), Gamma (Twist control 17), Delta (Twist control 23), and Omicron (Twist control 48) (Table 1). RNA was extracted from the virus panel and whole viruses using the Perkin Elmer Chemagic viral RNA extraction kit (Chemagic kit CMG-1033-S designed for SARS-CoV-2). RNA was used undiluted as the template in digital droplet PCR with mutation primer and probes (see further details on digital PCR below). The concentration of targets used in the *in vitro* specificity testing was approximately 275 copies per well. The mutation assays were challenged against the respiratory panel gRNA in single wells and nontarget variant gRNA in 8 replicate wells. Positive PCR controls (Table 1) were included on each plate.

The sensitivity and specificity of the mutation assays were further tested by diluting target variant gRNA (Table 1) for the mutations in no (0 copies), low (100 copies), and high (10,000 copies) backgrounds of WT gRNA. Each dilution was run in three replicate wells. The number of copies of variant mutation sequences input to each well was estimated using a dilution series of variant gRNA in no background; the vendor-specified concentration of the variant gRNA was scaled by the slope of the curve relating the measured ddRT-PCR concentration and the calculated input concentration based on the vendor estimates. Our experience suggests that vendor estimates can be imprecise. PCR negative controls were run in 4 wells per plate.

### Wastewater samples.

A publicly owned treatment work (POTW) that serves populations in Santa Clara County, California, USA (San José-Santa Clara Regional Wastewater Facility), was included in the study. It serves approximately 1,500,000 people; a further description of the POTW can be found in a report by Wolfe et al. (1).

Samples of approximately 50 mL of settled solids were collected by POTW staff using sterile technique in clean, labeled bottles. POTW staff manually collected a 24-h composite sample (21). Samples were immediately stored at 4°C, transported to the lab, and processed within 6 h of collection.

Samples were collected daily for a larger COVID-19 wastewater surveillance effort starting in November 2020 (1), and a subset of these samples are used in the present study and were chosen to span the period prior to and including presumed emergence of different variants. Generally, sampling was about once per week or month prior to presumed emergence and then 3 to 7 times per week during and after the period of emergence. Details on sampling frequency are provided in Table 3. A previous study (19) reported Alpha mutation data for the POTW, and those data are included in our analysis for completeness. That same study reported some Delta mutation data (*n* = 48, data until 1 August 2021) for the POTW, and those data are included here. The methods below describe those used for the new measurements, including those for Mu, Lambda, Beta/Gamma, Delta (measured daily between 1 August 2021 and 2 January 2022), and Omicron mutations.

**TABLE 3:** Details of sample collection for different assay applications^*a*^

Variant mutation	Frequency of sampling	*n*	Previously published?	No. of days RNA stored at −80°C for samples newly processed as part of this study
Mu	Biweekly: 1/21/21–3/30/21 Weekly: 4/1/21–5/25/21 3 per wk: 5/26/21–11/15/21	90	No	4–300
Beta/Gamma	One sample from 2/23/21 Weekly: 4/17/21–7/26/21	16	No	0–2
Delta	Biweekly to weekly: 2/7/21–5/1/21 3 per wk: 5/1/21–9/3/21 Daily: 9/4/21–11/30/21 3 per wk: 12/3/21–1/3/22	156	Partially, *n* = 48 collected between 2/7/21 and 7/30/21 (19)	0–30
Alpha	Monthly: 7/14/20–3/25/21 Daily: 3/28/21–8/8/21	133	Yes (19)	NA
Lambda	Weekly for 2 wks: 11/1/21 and 11/8/21	2	No	0–2
Omicron	Weekly: 11/2/21–11/23/21 3 per wk: 11/29/21–12/5/21 Daily: 12/6/21 and 1/2/22	35	No	0–30

a Details include frequency of sample collection for different assay applications, number of samples included in this study, whether any of the data have been published, and the time range that RNA samples were stored between extraction of RNA and running the PCR assays. RNA extraction occurred on the day of sample collection, as explained in Materials and Methods. Dates are reported as month/day/year.

RNA was extracted from the 10 replicate aliquots of dewatered settled solids as described elsewhere (1, 23, 24). This process includes dilution of the solids in DNA/RNA Shield (Zymo, Irvine, CA) as a means to alleviate inhibition (25). RNA was subsequently processed immediately (within 24 h of sample collection) to measure concentrations of the N gene of SARS-CoV-2, pepper mild mottle virus (PMMoV), and bovine coronavirus (BCoV) recovered using digital droplet RT-PCR methods described in detail elsewhere (1, 26). The N gene assay targets a region of the N gene that is conserved across these variants. PMMoV is highly abundant in human stool and wastewater globally (27, 28) and is used as an internal recovery and fecal strength control for the wastewater samples (29). BCoV was spiked into the samples and used as an additional recovery control; all samples were required to have greater than 10% BCoV recovery. RNA extraction and PCR negative and positive controls were included to ensure no contamination, as described by Wolfe et al. (1) The N gene measurement was multiplexed with the Delta mutation assay in samples processed after 1 August 2021, and the Omicron mutation assay was multiplexed in samples processed after 6 December 2021. For the other mutation assays, the extracted RNA was stored at –80°C for a period of time (Table 3) before it was analyzed for the N gene and the Mu, Beta/Gamma, or Lambda mutation assay in a multiplex digital RT-PCR assay. The SARS-CoV-2 N gene was run a second time for assays run on stored RNA to test for RNA degradation during storage at –80°C (no to minimal degradation was observed [see supplemental material]). Each of the 10 replicate RNAs extracted was run in its own well, and the 10 wells were merged for analysis. Wastewater data are available publicly at the Stanford Digital Repository (https://purl.stanford.edu/hs561fr5902); results below are reported as suggested in the EMMI guidelines for reporting ddRT-PCR measurements in environmental samples (30).

### ddRT-PCR.

Digital RT-PCR was performed on 20-μL samples from a 22-μL reaction volume, prepared using 5.5 μL template, mixed with 5.5 μL of supermix from the One-Step RT-ddPCR advanced kit for probes (Bio-Rad 1863021), 2.2 μL reverse transcriptase, 1.1 μL dithiothreitol (DTT), and primers and probes at a final concentration of 900 nM and 250 nM, respectively. Template was diluted 1:100 for measuring PMMoV and BCoV. Primers and probes were purchased from IDT (for sequences, see Table 3). Droplets were generated using the AutoDG automated droplet generator (Bio-Rad). PCR was performed using Mastercycler Pro with the following cycling conditions: reverse transcription at 50°C for 60 min, enzyme activation at 95°C for 5 min, 40 cycles of denaturation at 95°C for 30 s, and annealing and extension at 61°C (for SARS-CoV-2 targets) or 56°C (for PMMoV/BCoV targets) for 30 s, enzyme deactivation at 98°C for 10 min, and then an indefinite hold at 4°C. The ramp rate for temperature changes was set to 2°C/s, and the final hold at 4°C was performed for a minimum of 30 min to allow the droplets to stabilize. Droplets were analyzed using the QX200 droplet reader (Bio-Rad). All liquid transfers were performed using the Agilent Bravo (Agilent Technologies).

Thresholding was carried out using QuantaSoft analysis pro software (Bio-Rad; version 1.0.596). In order for a sample to be recorded as positive, it had to have at least 3 positive droplets.

For the wastewater samples, the concentrations of RNA targets were converted to concentrations per dry weight of solids in units of copies (cp)/g dry weight by using dimensional analysis. The dry weight of the dewatered solids was determined by drying (24). Using this approach, three positive droplets correspond to a concentration between ∼500 and 1,000 cp/g; the range in values is a result of the range in the equivalent mass of dry solids added to the wells. The total error is reported as standard deviations and includes the errors associated with the Poisson distribution and the variability among the 10 replicate wells.

### Variants present in regional clinical specimens.

The 7-day, centered, rolling average fraction of clinical specimens sequenced from the State of California and classified as Alpha, Beta, Gamma, Mu, Lambda, Delta, and Omicron as a function of specimen collection data were acquired through https://www.outbreak.info, which collates data from GISAID. Data were downloaded from https://www.outbreak.info on 5 January 2022 for all variants, except for Omicron, for which data were downloaded on 10 January 2022. Data were acquired in the form of time series plots, and data were extracted using PlotDigitizer (https://plotdigitizer.com/).

### Statistics.

We hypothesize that wastewater concentrations of characteristic variant mutations are associated positively with the proportion of infections caused by the variant in the contributing population. Because data on incidence rates of COVID-19 caused by specific variants at the sewershed level are not readily available, we used state-level data on the fraction of sequenced clinical specimens identified as specific variants to represent this variable. We normalized the wastewater concentration of the variant mutation by the concentration of the N gene to represent the fraction of total SARS-CoV-2 RNA (represented by the N gene assay target, which is conserved across variants) that comes from the variant; in the present article, this concentration is referred to as the relative concentration of the mutation. We applied a five-adjacent-sample-box average smoothing algorithm to the relative concentrations to aid in visualization but used raw data in statistical analyses. We used Kendall's tau (herein tau) to test for associations between the relative concentration and the fraction of clinical specimens assigned to the corresponding variant, as the two variables were not normally distributed (Shapiro-Wilk test, *P* < 0.05 for all). The measured relative concentration was matched to the 7-day, centered, rolling average fraction of clinical specimens classified as the associated variant obtained from https://www.outbreak.info.
