# Supplementary material for: Detection of SARS-CoV-2 Variants Mu, Beta, Gamma, Lambda, Delta, Alpha, and Omicron in Wastewater Settled Solids Using Mutation-Specific Assays Is Associated with Regional Detection of Variants in Clinical Samples
Source: Appl Environ Microbiol. 2022 Apr 5;88(8):e00045-22. doi: 10.1128/aem.00045-22 (PMC9040616; doi:10.1128/aem.00045-22)
Supplement: Supplemental file 1 — Text S1, Table S1, and Fig. S1. Download aem.00045-22-s0001.pdf, PDF file, 0.1 MB [file aem.00045-22-s0001.pdf]

Supporting Material for

Detection of SARS-CoV-2 variant Mu, Beta, Gamma, Lambda, Delta, Alpha, and Omicron in wastewater settled solids using mutation-specific assays is associated with regional detection of variants in clinical samples

Marlene Wolfe[a]*, Bridgette Hughes[b]*, Dorothea Duong[b], Vikram Chan-Herur[b], Krista R. Wigginton[c], Bradley J. White[c], Alexandria B. Boehm[d#]

a. Gangarosa Department of Environmental Health, Rollins School of Public Health, Emory University, Atlanta, GA, USA
b. Verily Life Sciences, South San Francisco, CA, USA
c. Civil & Environmental Engineering, University of Michigan, Ann Arbor, MI, USA
d. Civil & Environmental Engineering, Stanford University, Stanford, CA, USA

*co-first authors
#Address correspondence to: Alexandria Boehm, aboehm@stanford.edu

Minimal RNA degradation during storage at -80°C. RNA extracts were stored prior to analysis for the Mu, Delta, and Omicron mutations. The RNA extracts analyzed for the Mu mutation were stored for up to 300 days. The concentration of the SARS-CoV-2 N gene was measured immediately upon sample collection for a COVID-19 surveillance effort (1). It was measured again in multiplex with the assay targeting the Mu mutation after RNA samples were stored to gain insight into potential RNA degradation during storage. The concentration of SARS-CoV-2 RNA measured after storage at -80°C was not different from the concentration measured immediately after sample collection for the majority of the samples (42 of the 90 samples). For extracts in which the concentration was different, the median ratio of the measurements was 0.79 (average = 0.83) suggesting minimal RNA degradation during storage.

Seven RNA extracts were analyzed for the Delta mutation after being stored for up to 30 days. We conducted the same analysis as described above for these RNA extracts and found that the concentration of the N gene was not different after storage for 5 of the 7 extracts. For extracts in which the concentration was different, the median ratio of the measurements was 0.82 suggesting minimal RNA degradation during storage.

Seven RNA extracts were analyzed for the Omicron mutation after being stored for up to 30 days. We conducted the same analysis as described above for these RNA extracts and found that the concentration of the N gene was not different after storage for 4 of the 7 extracts. For extracts in which the concentration was different, the median ratio of the measurements was 0.81 suggesting minimal RNA degradation during storage.

Table S1.

Parameters used with primer design software.

- Product size ranges: 60-200
- Primer size: min 15, opt 20, max 36
- Primer melting temperature: min 50°C, optimal 60°C, max 65°C
- GC% content: min 40%, optimal 50%, high 60%
- concentration of divalent cations = 3.8 mM
- concentration of dNTPs needs to be 0.8 mM
- Internal Oligo: size min 15, optimal 20, max 27
- Internal Oligo: Melting temp min 62°C, optimal 63°C, max 70°C
- Internal Oligo: GC% min 30%, optimal 50%, max 80%

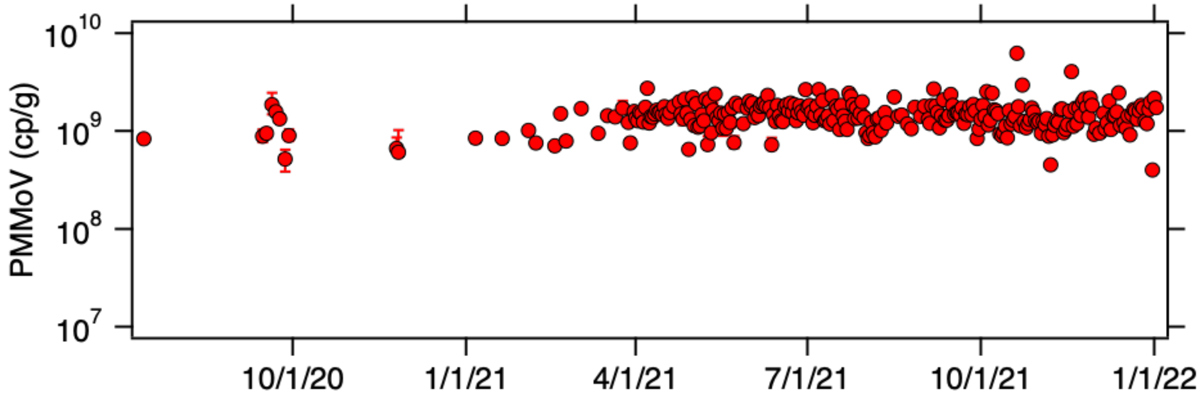

Figure S1. PMMoV RNA concentrations in units of cp/g for each sample used in this study. Error bars represent standard deviations and include poisson error and replicate well error and was output from the ddPCR machine software as “total error”. If the error bar cannot be seen on the marker, then it is smaller than the marker.

References

1. Wolfe MK, Topol A, Knudson A, Simpson A, White B, Duc V, Yu A, Li L, Balliet M, Stoddard P, Han G, Wigginton KR, Boehm A. 2021. High-Frequency, High-Throughput Quantification of SARS-CoV-2 RNA in Wastewater Settled Solids at Eight Publicly Owned Treatment Works in Northern California Shows Strong Association with COVID-19 Incidence. mSystems 0:e00829-21.
